# MST1/Hippo promoter gene methylation predicts poor survival in patients with malignant pleural mesothelioma in the IFCT-GFPC-0701 MAPS Phase 3 trial

**DOI:** 10.1038/s41416-019-0379-8

**Published:** 2019-02-11

**Authors:** Elodie Maille, Solenn Brosseau, Vincent Hanoux, Christian Creveuil, Claire Danel, Emmanuel Bergot, Arnaud Scherpereel, Julien Mazières, Jacques Margery, Laurent Greillier, Clarisse Audigier-Valette, Denis Moro-Sibilot, Olivier Molinier, Romain Corre, Isabelle Monnet, Valérie Gounant, Alexandra Langlais, Franck Morin, Guénaëlle Levallet, Gérard Zalcman

**Affiliations:** 1 Normandie Université, UNICAEN, INSERM, UMR 1086 ANTICIPE, Caen, France; 2 Normandie Université, UNICAEN, CEA, CNRS, ISTCT/CERVOxy group, GIP CYCERON, Caen, France; 3 Department of Thoracic Oncology & CIC1425, Hôpital Bichat-Claude Bernard, Assistance Publique Hôpitaux de Paris, Université Paris-Diderot, Paris, France; 4 Normandie Université, UNICAEN, UPRES-EA 2608, Caen, France; 5 Biomedical Research Unit, CHU de Caen, Caen, France; 6 Department of Pathology, Hôpital Bichat-Claude Bernard, AP-HP, Université Paris-Diderot, Paris, France; 7 Department of Pulmonology & Thoracic Oncology, CHU de Caen, Caen, France; 8 Department of Pulmonary and Thoracic Oncology, Centre Hospitalier Universitaire Lille, University of Lille, U1019 INSERM, Center of Infection and Immunity of Lille, Lille, France; 9 Department of Pulmonology, Hôpital Larrey, University Hospital of Toulouse, Toulouse, France; 10 Medical Oncology Department, Gustave Roussy, Villejuif, France; 11 Department of Multidisciplinary Oncology and Therapeutic Innovations, Assistance Publique Hôpitaux de Marseille, Aix Marseille University, Marseille, France; 12 Centre de Recherche en Cancérologie de Marseille (CRCM), INSERM UMR1068, CNRS UMR7258, Aix-Marseille University, UM105 Marseille, France; 13 Department of Pulmonology, Centre Hospitalier Toulon Sainte-Musse, Toulon, France; 14 Pôle Thorax et Vaisseaux, Centre Hospitalier Universitaire Grenoble, Grenoble, France; 15 Department of Pulmonology, Centre Hospitalier Le Mans, Le Mans, France; 16 Department of Pulmonology, Pontchaillou University Hospital, Rennes, France; 17 Department of Pulmonology, Centre Hospitalier Intercommunal Créteil, Créteil, France; 18 Department of Pulmonology, Hôpital Tenon, Assistance Publique Hôpitaux de Paris, Paris, France; 19 Intergroupe Francophone de Cancérologie Thoracique (IFCT), Paris, France; 20 Department of Pathology, CHU de Caen, Caen, France; 21 U830 INSERM “Genetics and Biology of Cancers, A.R.T group”, Curie Institute, Paris, France

**Keywords:** Prognostic markers, Mesothelioma

## Abstract

**Background:**

The Mesothelioma Avastin Cisplatin Pemetrexed Study (MAPS/NCT00651456) phase 3 trial demonstrated the superiority of bevacizumab plus pemetrexed–cisplatin triplet over chemotherapy alone in 448 malignant pleural mesothelioma (MPM) patients. Here, we evaluated the prognostic role of Hippo pathway gene promoter methylation.

**Methods:**

Promoter methylations were assayed using methylation-specific polymerase chain reaction in samples from 223 MAPS patients, evaluating their prognostic value for overall survival (OS) and disease-free survival in univariate and multivariate analyses. MST1 inactivation effects on invasion, soft agar growth, apoptosis, proliferation, and YAP/TAZ activation were investigated in human mesothelial cell lines.

**Results:**

STK4 (MST1) gene promoter methylation was detected in 19/223 patients tested (8.5%), predicting poorer OS in univariate and multivariate analyses (adjusted HR: 1.78, 95% CI (1.09–2.93), *p* = 0.022). Internal validation by bootstrap resampling supported this prognostic impact. MST1 inactivation reduced cellular basal apoptotic activity while increasing proliferation, invasion, and soft agar or in suspension growth, resulting in nuclear YAP accumulation, yet TAZ cytoplasmic retention in mesothelial cell lines. YAP silencing decreased invasion of MST1-depleted mesothelial cell lines.

**Conclusions:**

MST1/hippo kinase expression loss is predictive of poor prognosis in MPM patients, leading to nuclear YAP accumulation and electing YAP as a putative target for therapeutic intervention in human MPM.

## Background

Malignant pleural mesothelioma (MPM) is a rare yet aggressive cancer with poor prognosis mainly caused by occupational asbestos exposure.^1^ Recently, the Mesothelioma Avastin Cisplatin Pemetrexed Study (MAPS) demonstrated the benefit of bevacizumab plus cisplatin/pemetrexed doublet combination on both overall (OS) and progression-free survival (PFS) in 448 MPM patients.^2^ Following this trial, a biological study (Bio-MAPS) is currently evaluating new biomarkers, including the Hippo mammalian sterile 20-like kinase (MST1), as MPM has recently been proven to harbour frequent Hippo pathway alterations.^3^

MST1 (also named STK4), encoded by the human orthologue of the *Drosophila melanogaster* Hippo gene, and MST2 (STK3) are the best characterised of the five MST kinases (MST1/2/3/4 and YSK1) existing in mammals. MST1/2 primary function is to activate/phosphorylate the large tumour suppressor homologue 1/2 (LATS1/2) kinases, which in turn regulate the Yes-associated protein (YAP) and transcriptional co-activator with PDZ-binding motif (TAZ) activities. Inactive/phosphorylated YAP/TAZ are sequestered in the cytoplasm by proteins such as 14.3.3 or beta-catenin, while active/dephosphorylated YAP/TAZ interact with numerous transcription factors and cause transcription of genes involved in cell motility, growth, proliferation, and apoptosis.^4^ Thus, MST1 or MST2 loss results in hyperproliferation and tumourigenesis, commonly negated by YAP inactivation.^5^ MST1/2 kinases were also reported as contributing to the regulation of (i) apoptosis by establishing a complex with RASSF1A and CNK1 proteins^6,7^ or with the apoptosis-inhibiting protein kinase CK2,^8^ (ii) cell-cycle progression by catalysing the mitotic phosphorylation of MOBKL1A/1B,^9–13^ and (iii) migration/invasion processes by stabilising lamellipodial F-actin.^14–16^

Several studies suggest that Hippo signalling pathway deregulation is involved in pleural carcinogenesis, since the RASSF1A tumour suppressor gene, an upstream negative regulator of the pathway, is frequently methylated and inactivated in MPM.^17,18^ In our study, we first established a correlation between MST1 kinase gene promoter methylation and reduced OS in MPM patients. By transfecting cells with RNAi-MST1, we further reported that MST1 inactivation increases proliferation, invasion, and cell colony formation of MPM cell lines while decreasing their basal apoptotic activity. Finally, we demonstrated that the effect of MST1 expression loss depends on inappropriate activation of YAP.

## Methods

### Patients from the MAPS trial

From 13 February 2008 to 5 January 2014, 448 patients were randomly assigned to one of two treatments (223 (50%) to pemetrexed plus cisplatin and bevacizumab and 225 (50%) to pemetrexed plus cisplatin). Specific informed consent was obtained for the biological studies (Bio-MAPS), and the trial was approved by the appropriate ethics committee (CPP Ref 2007-20 Nord-Ouest III, France).

### DNA extraction and methylation-specific PCR assay

DNA samples from MPM were obtained from paraffin-embedded tumour tissue using the QIAamp DNA FFPE Tissue kit (Qiagen). Genomic DNA bisulphite modification was performed using the Epitect kit (Qiagen), according to the manufacturer’s instructions and as previously described.^19^ Polymerase chain reaction (PCR) was conducted with specific primers for either the methylated or unmethylated alleles (Table S1) in standard conditions for the following genes encoding proteins of the Hippo pathway or RASSF superfamily: RASSF1A, RASSF2A, RASSF5, RASSF10, MST1 (Figure S1), MST2, LATS1, and LATS2. RASSF6 methylation status was determined by the COBRA technique, as previously described.^20^

### Cell culture and transfection

Human MPM cell lines MSTO-211H, NCI-H2452, NCI-H28, and NCI-H2452 from the American Tissue Culture Collection (ATCC) were maintained in RPMI-1640 medium supplemented with 10% fetal bovine serum, 10 mM l-glutamine and streptavidin/penicillin, and kanamycin (100 µg/ml). Cells were transfected using JetPRIME^®^ (Polyplus-transfection^®^) with small-interfering ribonucleic acid (siRNA) or plasmid DNA, as listed in Table S2.

### Reverse transcription-quantitative real-time PCR

After RNA extraction, reverse transcription-quantitative real-time PCR (RT-qPCR) was performed with primer sets (Table S3) as previously described.^21^ RT-qPCR data were normalised to the human glyceraldehyde-3-phosphate dehydrogenase (GAPDH). Relative quantification was calculated using the delta-delta-Ct method.

### Immunoblotting

Whole-cell protein extracts were prepared as previously described,^22^ and proteins were detected by immunoblotting with the primary antibody from cell signalling (E-Cadherin, MST1, YAP/TAZ, P-Ser127YAP, GAPDH, vimentin), diluted to 1:1000 in Tween (0.1%)–tris-buffered saline buffer and horseradish peroxidase-conjugated secondary antibody, and then revealed by enhanced chemiluminescence using the ECL kit (Promega™). Densitometry results of western blot were analysed with ImageJ software. The signal intensity of each band was normalised with GAPDH densitometry values.

### Immunofluorescence and image analysis

Transfected cells were fixed and permeabilised as previously described.^20^ The primary antibodies were YAP (Cell Signaling, 1/150), TAZ (Cell Signaling, 1/150), alpha-tubulin (Sigma Aldrich, 1/300), actin (Cell Signaling, 1/300), Fascin (Cell Signaling, 1/300) or cytochrome *C* (BD Biosciences, 1/50). The AlexaFluo633, AlexaFluo555 or 488-labelled (Invitrogen™) secondary antibodies were added for 1 h. Coverslips were mounted with 4′,6-diamidino-2-phenylindole (DAPI; Santa Cruz^TM^) and images captured using high-throughput confocal microscopy (FluoView FV1000, Olympus™).

### Quantification and measure of cell cytoplasmic extensions

After immunolabeling tubulin filaments, number and length of cytoplasmic extensions from almost 200 cells were assayed from 10 images captured randomly at 20× objective with a high-throughput confocal microscopy (FluoView FV1000, Olympus™) using ImageJ software (version 1.50d).

### Invasion

A total of 15 × 10^3^ cells were added in serum-free medium to the top invasion chambers of 24-well transwell plates containing cell culture insert with 8 μm pores (BD BioCoat Matrigel^®^ Invasion Chamber, BD Biosciences™). Complete media supplemented with hepatocyte growth factor were added to the bottom chambers. Cells were incubated for 48 h and then removed; migrating (bottom) cells were stained with crystal violet.

### Soft agar assay

Base agar matrix (100 µl, Cell Biolabs) was seeded in a 96-well plate and 1500 cells/well layered on agar followed by 50 µl of 2× complete medium. After 25 days, colonies were stained and counted for each well.

### Spheroid culture

At 24 h post transfection, cells were reseeded with complete medium in 24-well plates without adhesion (Nunclon™ Sphera™ Microplates, Thermo Fisher Scientific). Sphere formation was evaluated on day 6 at ×10 magnification with a phase-contrast inverted microscope.

### Apoptosis measurement

DNA fragmentation and Caspase 3/7 activation were assayed using the Cell Death Detection ELISA plus kit (Roche, USA) and the Caspase-Glo 3/7 Luminescence Assay (Promega Corp. Madison, WI, USA), respectively, according to the manufacturer’s instructions.

### Bromodeoxyuridine incorporation

Cells were transfected, left for 24 h, then labelled with bromodeoxyuridine (BrdU) (1:500 dilution, cell proliferation assay, Millipore) for a further 24 or 48 h. BrdU incorporation was measured according to the manufacturer’s instructions.

### Statistical analysis

The Bio-MAPS study was a pre-planned ancillary and exploratory study. The characteristics of patients with promoter methylation analysis were compared to those without using chi-squared tests or Fisher’s exact tests for qualitative variables, and Student’s *t*-tests or Mann–Whitney tests for quantitative variables.

The prognosis value of MST1 promoter hypermethylation was assessed for PFS and OS using univariate and multivariate Cox models. Cofactors introduced to the multivariate model were: treatment arm, stratification factors used in the MAPS trial (histology, performance status (PS), and smoking status), and clinical factors known to be associated with survival (gender, age, sex, haemoglobin, white blood cells, and platelets).

Hazard ratios (HRs) were estimated with their 95% confidence intervals (95% CIs). Bootstrap resampling was used to assess the model’s stability and the optimism-corrected concordance index (c-index). The data were analysed with IBM SPSS software Version 22.0.

In vitro data are presented as means ± SEM; each experiment was performed at least three times independently. Statistical differences were determined either by one-way analysis of variance (ANOVA) or Dunnett’s multiple comparison test to compare each experiment’s condition with siMST1 (GraphPad Software, Inc. USA). Statistical significance was set at *p* ≤ 0.05.

## Results

### MST1 promoter hypermethylation predicts shorter OS of MPM patients

Assessing the baseline characteristics of patients from the MAPS trial reveals that biological parameters (haemoglobin, platelet count, leucocyte count), general status (PS 0–1 versus >2), and histology (epithelioid vs. sarcomatoid/mixed) are potent prognostic factors of OS (*p* < 0.05) and PFS in univariate and multivariate analyses (2) in this subset of MAPS patients. The baseline characteristics of the 223 patients in whom gene methylation assays were performed did not differ from those of patients for whom such analyses were not possible (Table S4). RASSF1A, RASSF2A, RASSF6, and RASSF10 were found to be methylated in 11.1%, 14.5%, 21.5%, and 4.4% of samples, respectively, while no sample exhibited any RASSF5, MST2, LATS1, or LATS2 methylation. None of these methylations influenced survival in univariate analysis (data not shown).

**Fig. 1:**
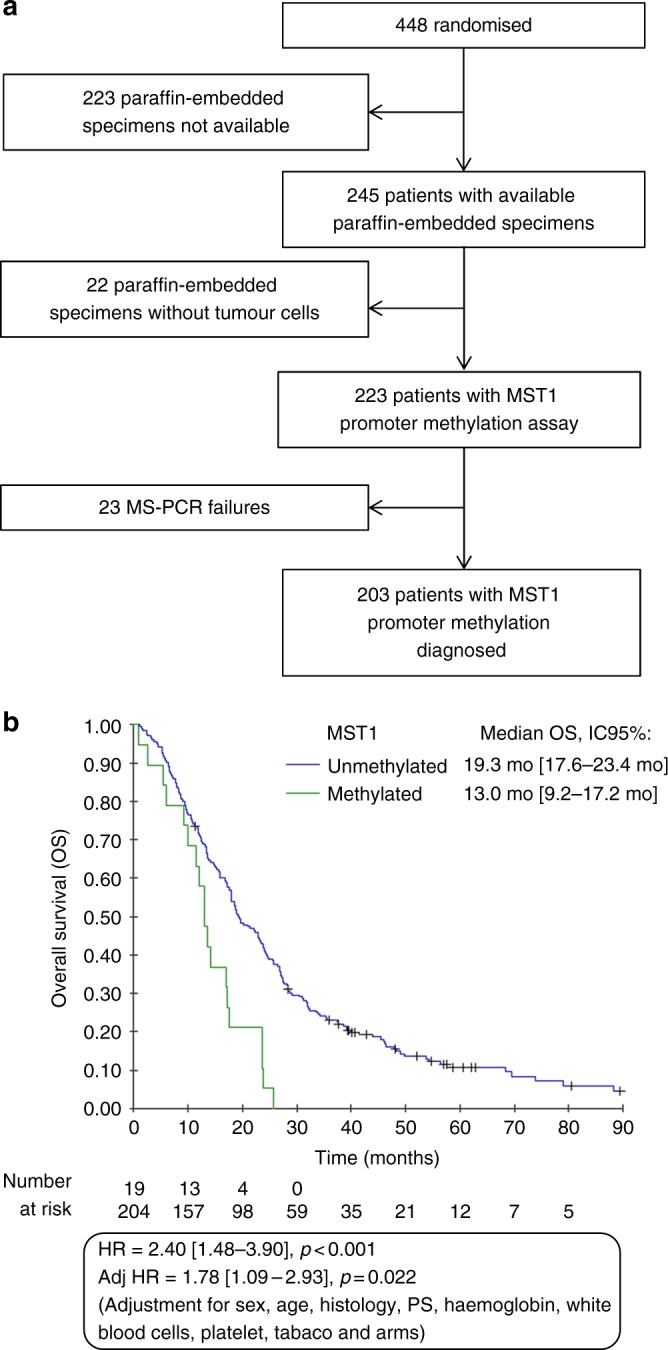
**a** Flow chart. **b** Kaplan–Meier curve of overall survival according to the MST1 promoter status (methylated or unmethylated)

MST1 promoter methylation status was available for 223/448 patients from MAPS (Fig. 1a), and MST1 promoter was methylated in 19/223 samples (8.5%) (representative methylation-specific PCR (MS-PCR) are shown in Figure S1).

In univariate analysis, the median OS of patients with methylated MST1 promoter was 1.4 times lower than that of patients with unmethylated MST1 promoter (13.0 versus 19.3 months, HR: 2.40, 95% CI (1.48–3.90), *p* < 0.001) (Fig. 1b). Moreover, univariate analyses showed that methylation of MST1 gene predicted worse survival in each treatment group separately, with HR = 2.29 (95% CI 1.13–4.65), *p* = 0.021 for the chemotherapy arm and 2.50 (95% CI 1.27–4.90), *p* = 0.0079 for the bevacizumab arm (Figure S2).

This result was confirmed in multivariate analysis in the whole series, both treatment arms analysed together (adjusted HR: 1.78, 95% CI (1.09–2.93), *p* = 0.022, adjustment for gender, age, histology, PS, haemoglobin, leucocyte and platelet counts, smoking, and treatment arm) (Fig. 1b), and validated by a bootstrap procedure: MST1 methylation was significantly associated with worse OS in 59% of 1000 bootstrapped samples, with an optimism corrected c-index of 0.67.

When the multivariate analysis was performed separately in the two treatment arms, the survival impact only remained significant in the bevacizumab arm (HR: 2.37, (95% CI (1.16–4.83), *p* = 0.017). However, an adjusted interaction test did not support MST gene methylation predictive value, with adj. HR: 1.44 (95% CI 0.71–2.93), *p* = 0.38 (Figure S2).

Finally, MST1 inactivation did not significantly predict PFS for MPM patients, although there was a correlating trend of a deleterious impact (HR: 1.53, 95% CI (0.95–2.46), *p* = 0.082 in univariate analysis).

### MST1 depletion modifies human mesothelial cell stretching

Since MST1 methylation status was the only alteration found to significantly influence prognosis in our large series of MPM patients, we focussed our functional mesothelial cell studies on this protein’s role.

**Fig. 2:**
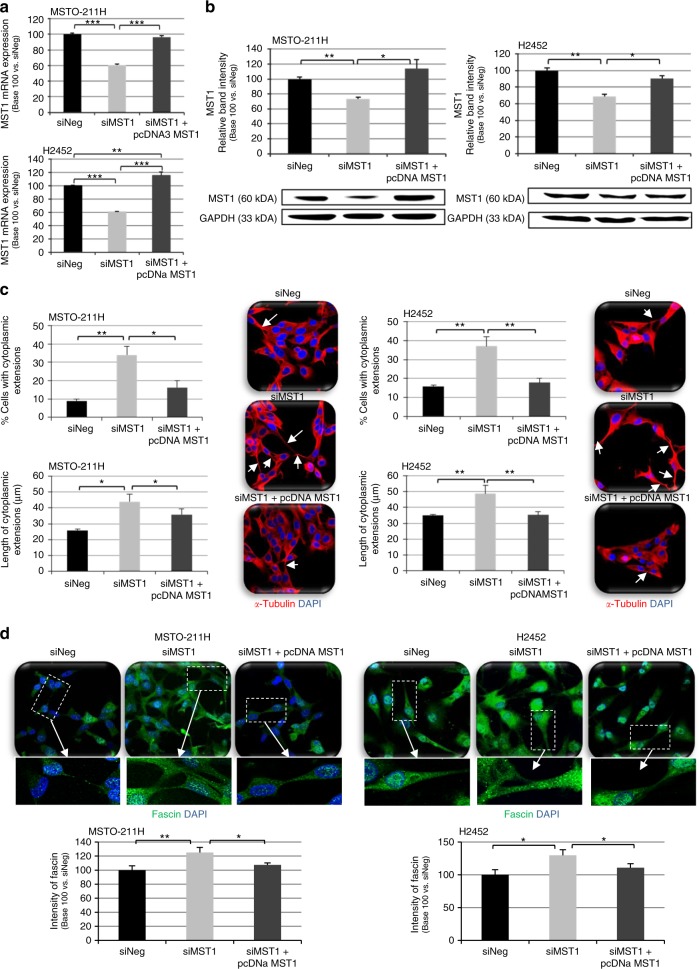
MST1 depletion causes morphological changes. **a**–**d** MST0-211H and H2452 cells were transfected with siNeg, siMST1, or siMST1+pcDNAMST1, analysed 48 h after transfection. Expression of MST1 was analysed by reverse transcription-quantitative real-time PCR (RT-qPCR) (**a**) and western blot (**b**), using glyceraldehyde-3-phosphate dehydrogenase (GAPDH) as an internal control. Quantification of number of cytoplasmic extensions and their size (µm) after α-Tubulin staining (**c**) and quantification of Fascin expression (**d**) by immunofluorescence and confocal microscopy in almost 200 cells using ImageJ software. Representative confocal pictures (**c**, **d**) are presented for cells stained for α-Tubulin (red), Fascin (green), and nuclei stained with 4′,6-diamidino-2-phenylindole (DAPI). For all histograms, error bars indicate the SEM of at least three independent experiments; *p < 0.05, **p < 0.01, and ***p < 0.001, using an analysis of variance (ANOVA) test followed by Dunnett’s test

MST1 was silenced in four human mesothelial cell lines (MSTO-211H, H2452, H28, and H2452) with normal basal MST1 expression (as evidenced by MS-PCR and qRT-PCR, data not shown), using siRNA-MST1. After testing several small interfering RNA (siRNA) sequences, we retained two that decreased both MST1 mRNA and protein expression by at least 40% in MPM cells (Fig. 2a, b, S3A). To validate the specificity of the effects observed in the absence of MST1, we have also introduced an experimental condition in which the cells are transfected with both a siMST1 and a plasmid carrying the coding sequence of MST1. The plasmid was brought at a concentration determined to overcome the amount of siRNA silencing the endogenous MST1 (Fig. 2a, b, S3A).

As evidenced by alpha-tubulin immunostaining, MST1-depleted cells were much more “stretched” than cells transfected with a control siRNA (siNeg). The shape of MSTO-211H or H2452 cells (Fig. 2c) (as H2052 and H28, data not shown) appeared significantly modified, and MST1 knockdown cells exhibited more cytoplasmic extensions than mock-transfected cells. Epithelial (E-cadherin) and mesenchymal (vimentin) marker quantification revealed that the morphological changes induced by MST1 loss were not caused by an epithelial–mesenchymal transition, since we found no typical expression switch between epithelial and mesenchymal markers (data not shown).

Cytoplasmic expansions from MST1-depleted cells were shown to be actual filopodia, as confirmed by the fascin enrichment in these structures (Fig. 2d), thus suggesting an increased ability for cell migration upon MST1 knockdown.

### MST1 loss increases invasion and growth in agar of MPM cells

**Fig. 3:**
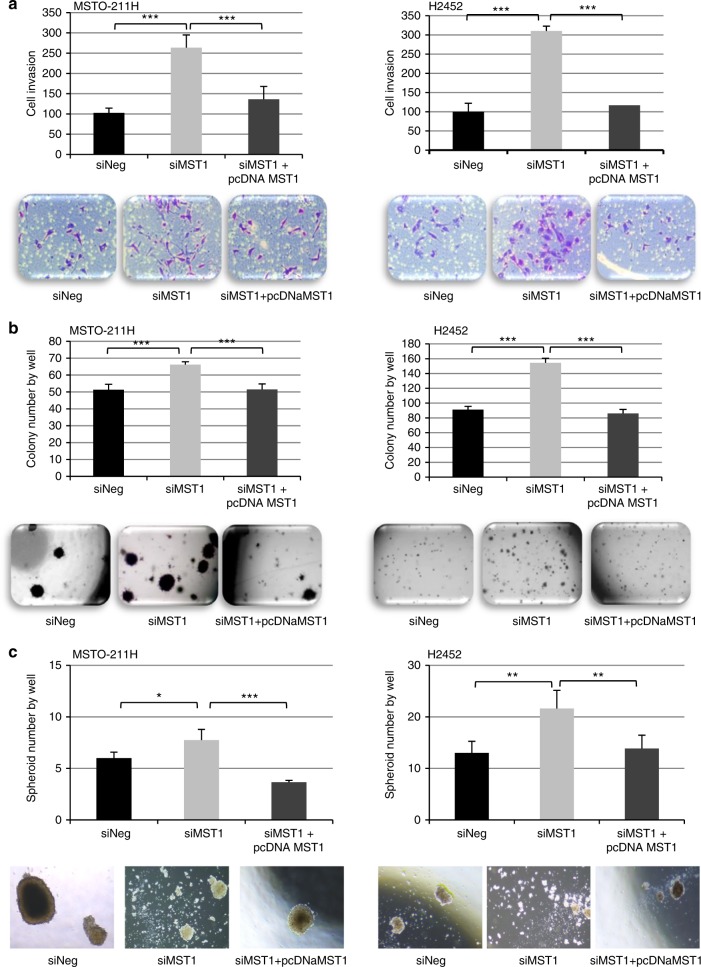
MST1 depletion increases cell invasion and anchorage-independent growth and decreases spheroid diameter. **a**–**c** MST0-211H and H2452 cells were transfected with siNeg, siMST1, or siMST1+pcDNAMST1, experiments were performed 48 h after transfection. **a** Invasion capacity of transfected cells on BioCoat Matrigel Invasion Chamber for 48 h. **b** Quantification of colonies in soft agar after 21 days. **c** Quantification of spheroid after 6 days. Representative picture is provided under histograms. For all histograms, error bars indicate the SEM of at least four independent experiments; *p < 0.05, **p < 0.01, and ***p < 0.001, using an analysis of variance (ANOVA) test followed by Dunnett’s test

We observed that MST1 depletion significantly increased the ability of MSTO-211H, H2452 (Fig. 3a), H28, and H2052 (Figure S3B) cells to invade Matrigel^®^. Moreover, colony formation on soft agar was increased by MST1 depletion in MSTO-211H, H2452 (Fig. 3b), H28, and H2052 (Figure S3C).

Finally, by impeding substrate-cell attachment, we caused spheroid formation increase in MSTO-211H and H2452 cells in absence of the MST1 kinase (Fig. 3c). For MST1-depleted MSTO-211H cells, we also observed that the cells were more individualised than control cells, which necessarily group into cell clumps.

### MST1 depletion decreases basal apoptosis and increases proliferation of MPM cells

**Fig. 4:**
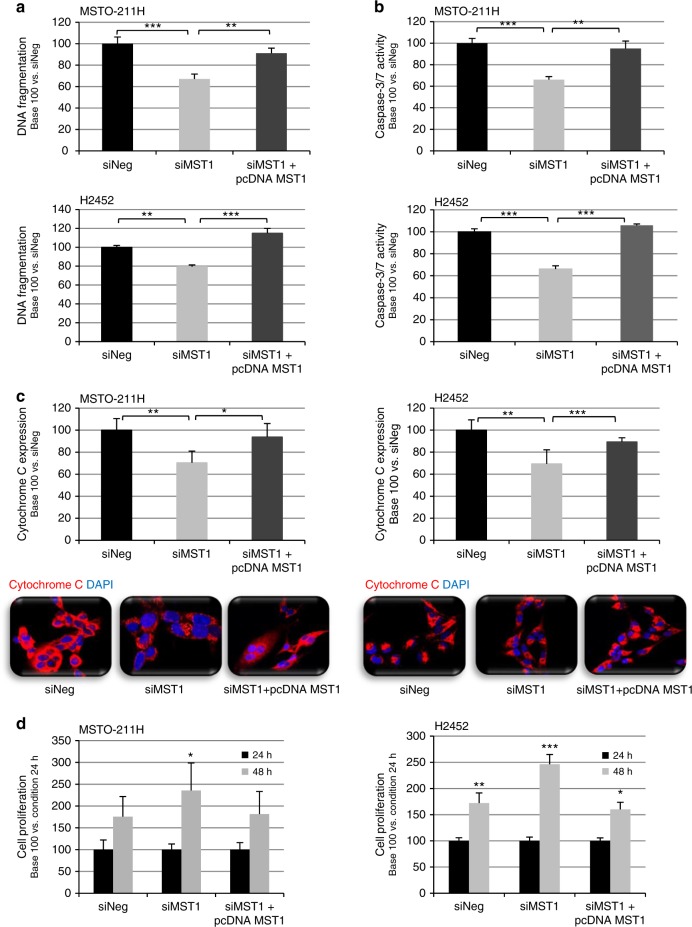
MST1 depletion decreases apoptosis and increases cell proliferation. **a**–**d** MST0-211H and H2452 cells were transfected with siNeg, siMST1, or siMST1+pcDNAMST1. Experiments performed 48 h after transfection. **a** Caspase 3/7 activity. **b** DNA fragmentation. **c** Quantification of cytochrome *c* (red) and representative immunofluorescent confocal picture, nuclei stained with 4′,6-diamidino-2-phenylindole (DAPI). **d** Cell proliferation assays. For all histograms, error bars indicate the SEM of at least three independent experiments; *p < 0.05, **p < 0.01, and ***p < 0.001, using an analysis of variance (ANOVA) test followed by Dunnett’s test

MSTO-211H and H2452 cell lines transfected with siMST1 presented significantly reduced DNA fragmentation (Fig. 4a), suggesting decreased apoptosis. This result was consistent with the decreased caspase 3/7 activity also measured in the absence of MST1 in the MSTO-211H and H2452 cells (Fig. 4b), as in H28 and H2052 cells (Figure S3D), and the lower intensity of cytochrome *C* staining quantified in MSTO-211H and H2452 cells (Fig. 4c), all assays exploring apoptosis ability.

This basal apoptotic activity decrease in MST1-depleted cells was accompanied by increased cell proliferation for MSTO-211H and H2452 cells (Fig. 4d), or H28 and H2052 cells (Figure S3E), as measured by BrDU incorporation.

### MST1 loss leads to nuclear YAP accumulation but TAZ cytoplasmic retention

MST kinases are known to activate LATS kinases,^4^ which in turn are able to phosphorylate YAP, inducing either YAP cytoplasmic sequestration or YAP proteasome degradation.^4^ It should be noted that in MSTO-211H cells, cells with LATS1 kinase inactivation,^23^ the Hippo pathway remains functional as demonstrated by the increase of phosphorylation on the serine 127 of YAP with increasing cell density (Figure S4A) and the concomitant decrease in intensity of nuclear YAP (Figure S4B), LATS1 inactivation being probably compensated by another NDR kinases.

**Fig. 5:**
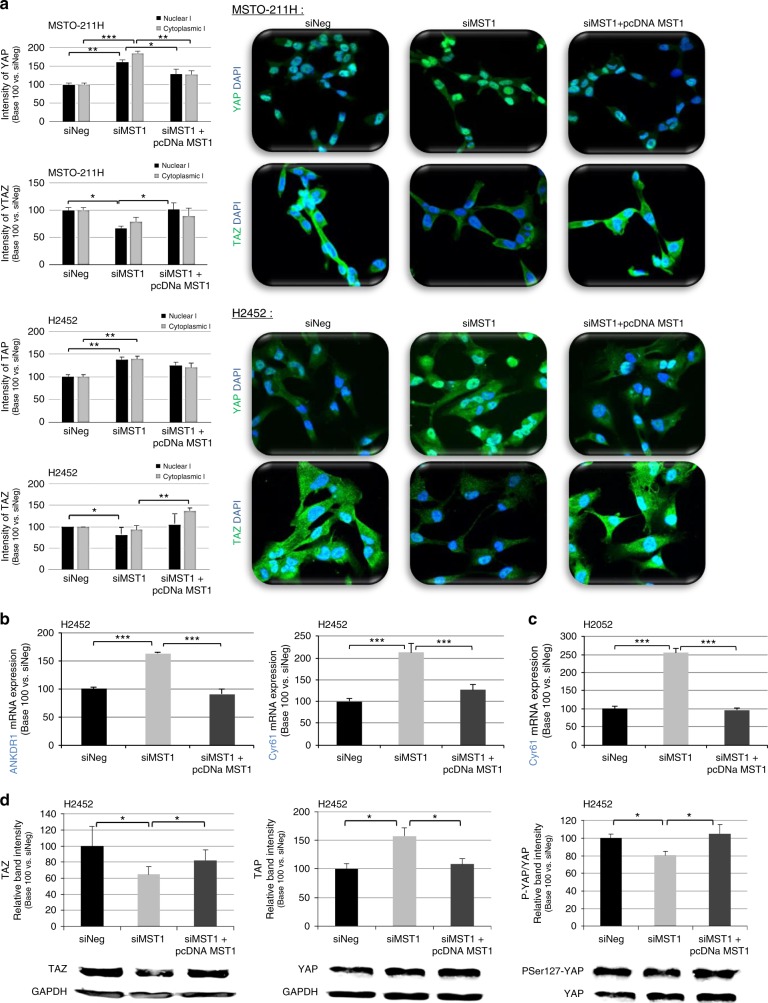
MST1 depletion increases nuclear Yes-associated protein (YAP) but decreases nuclear transcriptional co-activator with PDZ-binding motif (TAZ). **a**–**d** MST0-211H, H2452, and H2052 cells were transfected with siNeg, siMST1, or siMST1+pcDNAMST1. Experiments performed 48 h after transfection. **a** YAP nuclear and cytoplasmic localisation assayed by immunofluorescence. YAP/TAZ activity by quantifying ANKDR1 expression in **b** H2452 or **c** H2052 cells. **d** Quantification of TAZ, YAP, and PSer127-YAP protein levels using glyceraldehyde-3-phosphate dehydrogenase (GAPDH) as internal control. For all histograms, error bars indicate the SEM of at least three independent experiments; *p < 0.05, **p < 0.01, and ***p < 0.001, using an analysis of variance (ANOVA) test followed by Dunnett’s test

By using immunocytochemistry, we actually found that MST1 depletion led to nuclear YAP accumulation, suggesting a decrease in YAP phosphorylation. Conversely, using specific TAZ antibodies, we observed a decrease in nuclear TAZ, a YAP close homologue, in MSTO-211H and H2452 mesothelial cells (Fig. 5a).

We evaluated YAP/TAZ transcriptional activity by quantifying the expression of YAP/TAZ target genes: *CTGF, ANKDR1*, and *Cyr61*. *CTGF* expression did not significantly vary upon MST1 depletion (data not shown), while *ANKDR1* and *Cyr61* expression actually significantly increased by 1.6- to 2.5-fold, as shown in H2452 (Fig. 5b) and H2052 cells (Fig. 5c).

Western blot analyses further confirmed that YAP protein was more expressed in MST1-depleted MPM cell lines, while TAZ protein was less expressed. Furthermore, YAP protein should accumulate under its active form as evidenced by the P-Ser127YAP decrease detected in these cells, this form being reputed to be inactive (Fig. 5d).

**Fig. 6:**
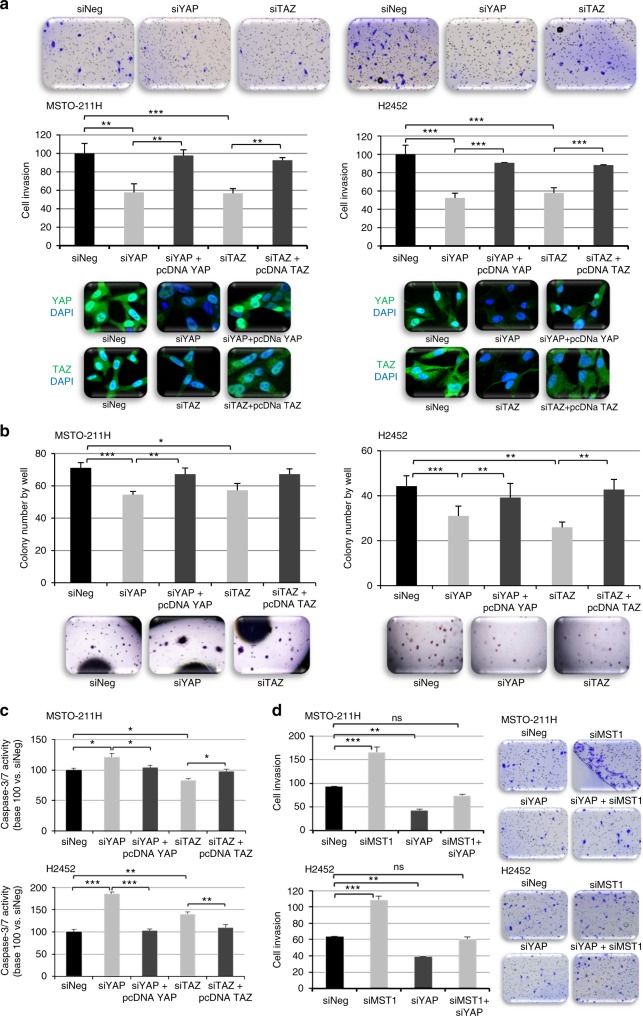
Yes-associated protein (YAP) or transcriptional co-activator with PDZ-binding motif (TAZ) depletion decreases cell invasion and anchorage-independent growth while increasing apoptosis. **a**–**d** MSTO-211H and H2452 cells were transfected with siNeg, siYAP, siTAZ, and siMST1 in combination or not with pcDNA YAP or TAZ. Experiments performed 48 h after transfection. **a** Invasion capacity of transfected cells on BioCoat Matrigel Invasion Chamber; representative image of YAP and TAZ extinction shown under the histograms. **b** Quantification of colonies in soft agar after 21 days. Representative picture is provided above (**a**) or under (**b**) histograms. **c** Caspase 3/7 activity. **d** Invasion capacity of transfected cells on BioCoat Matrigel Invasion Chamber for 48 h. For all histograms, error bars indicate the SEM of at least three independent experiments; *p < 0.05, **p < 0.01, and ***p < 0.001, using an analysis of variance (ANOVA) test followed by Dunnett’s test

We next evaluated apoptosis activity, invasion, and colony formation of MPM cell lines in the absence of either YAP or TAZ (Fig. 6, Figure S5). We found that YAP and TAZ extinction actually decreased MPM cell invasion (Fig. 6a, Figure S5,A) and colony numbers (Fig. 6b, Figure S5,B), and increased caspase 3/7 activity (Fig. 6c, Figure S5,C). Thus, the aggressive cell phenotype induced by MST1 loss could result from YAP cytoplasmic-to-nuclear shuttle and TAZ cytoplasmic retention. By silencing YAP in MST1-depleted MSTO-211H or H2452 cells, in line with such hypothesis, we were finally able to decrease their invasion through Matrigel^®^ (Fig. 6d).

## Discussion

Today, there is still no oncogenic “driver” identified in MPM enabling targeted therapeutics to be developed.^24^ To identify such a putative driver, the Bio-MAPS study set out to characterise the molecular abnormalities in tumours from patients enroled in the MAPS phase 3 trial. Focusing on Hippo pathway alterations, we assayed MST1 gene promoter hypermethylation, for the first time to our best knowledge, in a subset (8.5%) of MPM patients. MST1 promoter hypermethylation had been previously described in not only other cancers such as sarcoma^18^ and head and neck squamous cell carcinomas,^25^ but also in non-cancer diseases like autoimmune pancreatitis and rheumatoid arthritis.^26^ While MST1 inactivation had not previously been evidenced in MPM, our findings are in line with the reports of frequent genetic alterations of Hippo pathway members recently revealed in MPM^23^ and support the concept that Hippo pathway alterations are key events in pleural carcinogenesis.^23,27,28^

Interestingly, our study further revealed that the hypermethylation of MST1 promoter is associated with significantly worse OS for MPM patients, which is consistent with its tumour suppressor function and biomarker potential status. Our findings are consistent with those already reported in the literature for other cancers, such as breast cancer,^29^ hepatocellular carcinoma,^30^ and colorectal cancer.^31^

By mimicking MST1 loss in mesothelioma cell lines via MST1 RNA interference (RNAi) transfection, we demonstrated that MST1 prevented nuclear YAP accumulation while permitting TAZ cytoplasmic retention in mesothelial cells, thus inhibiting cell motility, growth without anchorage, and proliferation, while controlling basal apoptosis. These results are consistent with data from the literature, where MST1 role in invasion, migration, apoptosis, and cell proliferation has already been documented in various cancer models,^15,16,32–35^ though not yet in MPM. Moreover, MST1 or MST2 loss is known to lead to hyperproliferation and tumourigenesis, which are commonly prevented by concurrent YAP inactivation.^5^ Conversely, MST1 overexpression induces apoptosis, inhibits proliferation and tumour growth, and leads to YAP phosphorylation on Ser127 (thus YAP inactivation), subsequently provoking CTGF, AREG, and survivin (YAP-target genes) downregulation in hepatocellular carcinoma^36^ and non-small cell lung cancer.^37^ In line with such reports, our study found that the nuclear YAP accumulation induced by MST1 depletion is consistent with a P-Ser127YAP decrease in MST1-depleted cells with *ANKDR1* and *Cyr61* gene transcription increase, supporting the fact that YAP accumulated under its active form in the nucleus. We also revealed that YAP inactivation in MST1-depleted cells was able to fully reverse their ability to invade Matrigel^®^. The increase in nuclear YAP in the absence of MST1 could explain the poorer OS in patients with MPM and the more “aggressive” phenotype of mesothelial cells at the cellular level. That loss of MST1 expression involving YAP has already been reported in the literature,^34^ as has the involvement of YAP in the tumour progression of MPM, yet our study was the first to identify Hippo kinase inactivation leading to YAP deregulation.

YAP has been evidenced as a potential therapeutic target in MPM patients.^25,38,39^ In line with our results, another team recently reported that YAP inactivation in the MSTO-211H cells, which contain an inactive kinase-truncated LATS1 fusion protein and thus a constitutively nuclear active YAP by wild-type LATS1 kinase overexpression leading to YAP phosphorylation, was able to restore YAP to its cytoplasmic location, consisting of a surrogate marker for its functional inactivation . In such YAP-inactivated MSTO-211H cells, adherent or anchorage-independent cell growth was suppressed, as was colony formation. Similarly, another team recently experimented with H2452 cells with wild-type LATS1, using YAP RNAi-mediated knockdown, demonstrating that such YAP depletion also inhibited the growth of H2452 mesothelial cells and significantly suppressed Hippo pathway transcriptional activity, with decreased invasion, tumorosphere formation, and impaired stem cell self-renewal capacity.^38^ These data, along with our own findings, support the putative therapeutic potential of YAP inhibition in MPM.

Routinely performing MST1 methylation assay could thus help identify a subset of patients with poorer prognosis who could derive benefit from therapies targeting YAP, for instance by impairing the interaction of YAP with its transcriptional partners (e.g., TEAD 1–4 proteins). Our results, obtained in this homogeneous large series of MPM patients, all treated in a large phase 3 trial, confirm that targeting YAP in MPM represents a new and attractive therapeutic intervention in this disease, by targeting the actual features of MPM aggressiveness, responsible for unsatisfactory survival even under modern triplet regimens such as that used in the experimental arm of the MAPS trial, associating chemotherapy with the anti-angiogenesis drug bevacizumab.

## Supplementary information

TableS1

TableS2

TableS3

TableS4

Figures Legends

FigureS1

FigureS2

FigureS3

FigureS4

FigureS5

## Data Availability

All data are stored at the IFCT centre and can be made available upon request.
