# Supplementary material for: MST1/Hippo promoter gene methylation predicts poor survival in patients with malignant pleural mesothelioma in the IFCT-GFPC-0701 MAPS Phase 3 trial
Source: Br J Cancer. 2019 Feb 11;120(4):387–97. doi: 10.1038/s41416-019-0379-8 (PMC6461894; doi:10.1038/s41416-019-0379-8)
Supplement: Supplementary file 1 — TableS1 [file 41416_2019_379_MOESM1_ESM.docx]

**Table S1**. Methylation-specific polymerase chain reaction primer sequences.

|  | **Sequence (5’⭢3’)** | **T_M_** | **Size (bp)** | **Reference** |
| --- | --- | --- | --- | --- |
| ***LATS1 (Genbank access:*** [***NW_001838990.2***](http://www.ncbi.nlm.nih.gov/mapview/maps.cgi?maps=blast_set&db=allcontig_and_rna&na=1&gnl=ref%7CNW_001838990.2%7CHs6_WGA383_36&gi=157812010&term=157812010%5Bgi%5D&taxid=9606&RID=DFG3GKNE01N&QUERY_NUMBER=1&log$=nucltop)***)*** | | | | |
| **U:** | **F:** TGAATGATTAGAGTTGTGGGTGATGT | 60°C | 128 | Seidel *et al.,* 2007 |
|  | **R:** AAACATTTCCCAACATCACTTACACA |  |  |  |
| **M:** | **F:** GAACGATTAGAGTTGCGGGCGAC | 62°C | 126 |  |
|  | **R:** AACATTTCCCGACGTCGCTTACG |  |  |  |
| ***LAST2 (Genbank access : 26524)*** | | | | |
| **U:** | **F:** GGTGTTTTGTTTGGATTGGTATGTGGTT | 60 °C | 141 | Seidel *et al.,* 2007 |
|  | **R:** CATCTTCCCAAAACACTCACACCACA |  |  |  |
| **M:** | **F:** TTCGTTCGGATTGGTATGCGGTC |  | 137 |  |
|  | **R:** CCATCTTCCCGAAACGCTCACG |  |  |  |
| ***MST1 (Genbank access : NW_001838666.1)*** | | | | |
| **U:** | **F:** TTTGTGGGGTGGGTTTAGGAGGTTTGT | 63°C | 125 | Seidel *et al.,* 2007 |
|  | **R:** AACCAATAACCCCTCACCAACACAACAA |  |  |  |
| **M:** | **F:** GCGGGGCGGGTTTAGGAGGTTC |  | 120 |  |
|  | **R:** CCAATAACCCCTCACCGACGC |  |  |  |
| ***MST2 (Genbank access :*** [***NT_008046.15***](http://www.ncbi.nlm.nih.gov/mapview/maps.cgi?maps=blast_set&db=allcontig_and_rna&na=1&gnl=ref%7CNT_008046.15%7CHs8_8203&gi=51467074&term=51467074%5Bgi%5D&taxid=9606&RID=DFKPVRAY013&QUERY_NUMBER=1&log$=nucltop)***)*** | | | | |
| **U:** | **F:** TTTTAAGTGGGAGGGAGATTTGTTGTGG | 61°C | 108 | Seidel *et al.,* 2007 |
|  | **R:** AAAAACCAAAACACCAACCAACCAAACC |  |  |  |
| **M:** | **F:** CGGGAGGGAGATTCGTCGCG | 63°C | 99 |  |
|  | **R:** AAACCGAAACACCGACCGACCG |  |  |  |
| ***RASSF1A (Genbank access : NC_000003)*** | | | | |
| **U:** | **F:** TTTGGTTGGAGTGTGTTAATGTG | 60°C | 108 | Schagdarsurengin *et al.,* 2002 |
|  | **R:** CAAACCCCACAAACTAAAAACAA |  |  |  |
| **M:** | **F:** GTGTTAACGCGTTGCGTATC | 62°C | 96 |  |
|  | **R:** AACCCCGCGAACTAAAAACGA |  |  |  |
| ***RASSF2 (Genbank access : NT_011387.8)*** | | | | |
| **U:** | **F:** AGTTTGTTGTTGTTTTTTAGGTGG | 63°C | 108 | Hesson *et al.,* 2005 |
|  | **R:** AAAAAACCAACAACCCCCACA |  |  |  |
| **M:** | **F:** GTTCGTCGTCGTTTTTTAGGCG |  |  |  |
|  | **R:** AAAAACCAACGACCCCCGCG |  |  |  |
| ***Nore1A/RASSF5(Genbank access : NC_000001)*** | | | | |
| **U:** | **F:** ATTTATATTTGTGTAGATGTTGTTTGGTAT | 63°C | 215 | Hesson *et al.,* 2004 |
|  | **R:** ACTTTAACAACAACAACTTTAACAACTACA |  |  |  |
| **M:** | **F:** CGTCGTTTGGTACGGATTTTATTTTTTTCGGTTC | 62°C | 202 |  |
|  | **R:** GACAACTTTAACAACGACGACTTTAACGACTACG |  |  |  |

Hesson LB, Bièche I, Krex D, Criniere E, Hoang-Xuan K, Maher ER, Latif F. Frequent epigenetic inactivation of RASSF1A and BLU genes located within the critical 3p21.3 region in gliomas. *Oncogene*. 2004 Mar 25;**23**(13):2408-19.

Hesson LB, Wilson R, Morton D, Adams C, Walker M, Maher ER, Latif F. CpG island promoter hypermethylation of a novel Ras-effector gene RASSF2A is an early event in colon carcinogenesis and correlates inversely with K-ras mutations. *Oncogene*. 2005 Jun 2;**24**(24):3987-94.

Schagdarsurengin U, Gimm O, Hoang-Vu C, Dralle H, Pfeifer GP, Dammann R. Frequent epigenetic silencing of the CpG island promoter of RASSF1A in thyroid carcinoma. *Cancer Res.* 2002 Jul 1;**62**(13):3698-701.

Seidel C, Schagdarsurengin U, Blümke K, Würl P, Pfeifer GP, Hauptmann S, Taubert H, Dammann R. Frequent hypermethylation of MST1 and MST2 in soft tissue sarcoma. *Mol Carcinog.* 2007 Oct;**46**(10):865-71.
