# Supplementary material for: MST1/Hippo promoter gene methylation predicts poor survival in patients with malignant pleural mesothelioma in the IFCT-GFPC-0701 MAPS Phase 3 trial
Source: Br J Cancer. 2019 Feb 11;120(4):387–97. doi: 10.1038/s41416-019-0379-8 (PMC6461894; doi:10.1038/s41416-019-0379-8)
Supplement: Supplementary file 2 — TableS2 [file 41416_2019_379_MOESM2_ESM.docx]

**TableS2.** siRNA and plasmids used in this study.

|  | siRNA sequences | Plasmid  (Addgene reference) |
| --- | --- | --- |
| MST1 | si1 : GCGGAGCCAAUACUAUGAU  si2 : GCGGAGCCAAUACUAUGAU | #12203 |
| YAP | si1 : UGAGAACAAUGACGACCAA  si2 : CCACCAAGCUAGAUAAAGA | #27370 |
| TAZ | si1 : AGGTACTTCCTCAATCACA  si2 : CUAGGAAGGCGAUGAAUCA | #32840 |
