# Supplementary material for: MST1/Hippo promoter gene methylation predicts poor survival in patients with malignant pleural mesothelioma in the IFCT-GFPC-0701 MAPS Phase 3 trial
Source: Br J Cancer. 2019 Feb 11;120(4):387–97. doi: 10.1038/s41416-019-0379-8 (PMC6461894; doi:10.1038/s41416-019-0379-8)
Supplement: Supplementary file 3 — TableS3 [file 41416_2019_379_MOESM3_ESM.docx]

**TableS3.** Primers used in this study for quantitative real-time polymerase chain reaction.

|  | Forward (5’- 3’) | Reverse (5’-3’) |
| --- | --- | --- |
| MST1 | gtagccagcaccatgactga | ttgccaaagctgttgatctg |
| YAP | GCCGGAGCCCAAATCC | GCAGAGAAGCTGGAGAGGAATG |
| TAZ | ACCCACCCACGATGACCCCA | GCACCCTAACCCCAGGCCAC |
| CTGF | aggagtgggtgtgtgacga | ccaggcagttggctctaatc |
| ANKDR1 | agtagaggaactggtcactgg | tgggctagaagtgtcttcagat |
| Cyr61 | TTCTTTCACAAGGCGGCACTC | AGCCTCGCATCCTATACAACC |
