# Supplementary material for: MST1/Hippo promoter gene methylation predicts poor survival in patients with malignant pleural mesothelioma in the IFCT-GFPC-0701 MAPS Phase 3 trial
Source: Br J Cancer. 2019 Feb 11;120(4):387–97. doi: 10.1038/s41416-019-0379-8 (PMC6461894; doi:10.1038/s41416-019-0379-8)
Supplement: Supplementary file 4 — TableS4 [file 41416_2019_379_MOESM4_ESM.docx]

**TableS4. Baseline characteristics of Bio-MAPS (patients with available MST1 MSP-PCR) and MAPS patients without MST1 analysis.**

|  | | Bio-MAPS  (n=223) | | MAPS  (n=225) | p-value |  |
| --- | --- | --- | --- | --- | --- | --- |
| Sex | Male (n, %) | 171 | (76.7%) | 167 (74.2%) | NS |  |
|  | Female | 52 | (23.3%) | 58 (25.8%) |  |  |
| Median age (years) | | 66.8 | (61.9-70.2) | 65.0 (61.2-70.0) | NS |  |
| Haemoglobin (g/L, mean+/-SD) | | 132 | (±17) | 131 (±16) | NS |  |
| leucocytes (x10^9^/L, mean+/-SD) | | 8.6 | (±2.8) | 8.3 (±2.5) | NS |  |
| Neutrophils (x10^9^/L, mean+/-SD) | | 6.1 | (±2.6) | 6.0 (±2.4) | NS |  |
| Platelets (x10^9^/L, mean+/-SD) | | 350.1 | (±133.7) | 341.8 (±128.2) | NS |  |
| Treatment Arm* | PC | 108 | (48.4%) | 117 (52.0%) | NS |  |
|  | PCB | 115 | (51.6%) | 108 (48.0%) |  |  |
| Histology | Epithelioid | 182 | (81.6%) | 181 (80.4%) |  |  |
|  | Sarcomatoid or Biphasic | 41 | (18.4%) | 44 (19.6%) | NS |  |
| ECOG Performance Status | 0-1 | 215 | (96.4%) | 218 (96.9%) | NS |  |
|  | 2 | 8 | (3.6%) | 7 (3.1%) |  |  |
| Smoking status | Never smokers | 94 | (42.2%) | 100 (44.4%) | NS |  |
|  | Smokers | 129 | (57.8%) | 125 (55.6%) |  |  |

Data are n (%), median (IQR), or mean (SD).

PCB=pemetrexed plus cisplatin plus bevacizumab. PC=pemetrexed pluscisplatin.

ECOG=Eastern Cooperative Oncology Group.

NS = Not significant.
