# Supplementary material for: MST1/Hippo promoter gene methylation predicts poor survival in patients with malignant pleural mesothelioma in the IFCT-GFPC-0701 MAPS Phase 3 trial
Source: Br J Cancer. 2019 Feb 11;120(4):387–97. doi: 10.1038/s41416-019-0379-8 (PMC6461894; doi:10.1038/s41416-019-0379-8)
Supplement: Supplementary file 5 — Figures Legends [file 41416_2019_379_MOESM5_ESM.docx]

**Legends.**

**Figure1**. **A) Flow chart. B) Kaplan-Meier curve of overall survival according MST1 promoter status** (methylated or unmethylated).

**Figure 2. MST1 depletion causes morphological changes.**

**(A-D).** MST0-211H and H2452 cells were transfected with siNeg, siMST1, or siMST1+pcDNAMST1, analyzed 48h after transfection. Expression of MST1 was analyzed by RT-qPCR **(A)** and western blot **(B)**, using GAPDH as an internal control. Quantification of number of cytoplasmic extensions and their size (µm) after α-Tubulin staining **(C)** and quantification of Fascin expression **(D)** by immunofluorescence and confocal microscopy in almost 200 cells using ImageJ software. Representative confocal pictures **(C-D)** are presented for cells stained for α-Tubulin (red), Fascin (green), and nuclei stained with DAPI. For all histograms, error bars indicate the SEM of at least three independent experiments. *P <0.05, **P <0.01, and ***P <0.001, using an ANOVA test followed by Dunnett’s test.

**Figure 3. MST1 depletion increases cell invasion and anchorage-independent growth and decreases spheroid diameter.**

**(A-C)** MST0-211H and H2452 cells were transfected with siNeg, siMST1, or siMST1+pcDNAMST1, experiments were performed 48h after transfection. **A)** Invasion capacity of transfected cells on BioCoat Matrigel Invasion Chamber for 48h. **B)** Quantification of colonies in soft agar after 21 days. **C)** Quantification of spheroid after 6 days. Representative picture is provided under histograms. For all histograms, error bars indicate the SEM of at least four independent experiments. *P <0.05, **P <0.01, and ***P <0.001, using an ANOVA test followed by Dunnett’s test.

**Figure 4. MST1 depletion decreases apoptosis and increases cell proliferation.**

**(A-D)** MST0-211H and H2452 cells were transfected with siNeg, siMST1, or siMST1+pcDNAMST1. Experiments performed 48h after transfection. **A)** Caspase 3/7 activity. **B)** DNA fragmentation. **C)** Quantification of cytochrome c (red) and representative immunofluorescent confocal picture, nuclei stained with DAPI. **D)** Cell proliferation assays. For all histograms, error bars indicate the SEM of at least three independent experiments. *P <0.05, **P <0.01, and ***P <0.001, using an ANOVA test followed by Dunnett’s test.

**Figure 5. MST1 depletion increases nuclear YAP but decreases nuclear TAZ.**

**(A-D)** MST0-211H, H2452, and H2052 cells were transfected with siNeg, siMST1, or siMST1+pcDNAMST1. Experiments performed 48h after transfection. **A)** YAP nuclear and cytoplasmic localization assayed by immunofluorescence. YAP/TAZ activity by quantifying ANKDR1 expression in **B**) H2452 or **C)** H2052 cells. **D)** Quantification of TAZ, YAP, and PSer127-YAP protein levels using GAPDH as internal control. For all histograms, error bars indicate the SEM of at least three independent experiments. *P <0.05, **P <0.01, and ***P <0.001, using an ANOVA test followed by Dunnett’s test.

**Figure 6. YAP or TAZ depletion decreases cell invasion and anchorage-independent growth while increasing apoptosis.**

**(A-D)** MSTO-211H and H2452 cells were transfected with siNeg, siYAP, siTAZ, siMST1 in combination or not with pcDNA YAP or TAZ. Experiments performed 48h after transfection. **A)** Invasion capacity of transfected cells on BioCoat Matrigel Invasion Chamber; representative image of YAP and TAZ extinction shown under the histograms. **B)** Quantification of colonies in soft agar after 21 days. Representative picture is provided above **(A)** or under **(B)** histograms. **C)** Caspase 3/7 activity. **D)** Invasion capacity of transfected cells on BioCoat Matrigel Invasion Chamber for 48h. For all histograms, error bars indicate the SEM of at least three independent experiments. *P <0.05, **P <0.01, and ***P <0.001, using an ANOVA test followed by Dunnett’s test.

**Figure S1. Representative visualization of MST1 promoter hypermethylation status assayed by MS-PCR.** *Lane U:* unmethylated sequence; *Lane M:* methylated sequence. C+M: positive control for methylation; C+U: positive control for unmethylation (lymphocyte DNA); Cneg: H_2_0 replaces bisulfonated DNA. L: ladder (25 bp).

**FigureS2**. **Kaplan-Meier curve of overall survival according MST1 promoter methylation or not in pemetrexed plus cisplatin doublet arm (A) or in pemetrexed/cisplatin plus Bevacizumab arm (B).**

**Figure S3. MST1 depletion increases cell invasion, anchorage-independent growth, and proliferation while decreasing H28 and H2052** **apoptosis.**

**(A-E)** H28 and H2052 cells were transfected with siNeg, siMST1, or siMST1+pcDNAMST1. Experiments performed 48h after transfection. MST1 expression was analyzed by western blot **(A)** using GAPDH as an internal control. **B)** Invasion capacity of transfected cells on BioCoat Matrigel Invasion Chamber. Representative picture is shown under histograms. **C)** Quantification of colonies in soft agar after 21 days. **D)** Caspase 3/7 activity. **E)** Cell proliferation assays. For all histograms, error bars indicate the SEM of at least three independent experiments. *P <0.05, **P <0.01, and ***P <0.001, using an ANOVA test followed by Dunnett’s test.

**Figure S4. YAP is inactivate by cell density in MSTO-211H cells.**

MSTO-211H cells were grown up to 30, 50 or 90% of confluence. **A)** A western-blot was performed using anti-PhosphoSer127-Yap or anti-YAP. **B**) Intensity of nuclear Yap according cell density. Representatives pictures are shown under histograms. For all histograms, error bars indicate the SEM of at least three independent experiments. *P <0.05, **P <0.01, and ***P <0.001, using an ANOVA test followed by Dunnett’s test.

**Figure S5. YAP or TAZ depletion decreases cell invasion and anchorage-independent growth while increasing apoptosis.**

**(A-C)** H28 and H2052 cells were transfected with siNeg, siYAP, siTAZ in combination or not with pcDNA YAP or TAZ. Experiments performed 48h after transfection. **A)** Invasion capacity of transfected cells on BioCoat Matrigel Invasion Chamber. Representative picture is shown above histograms. Representative image of YAP and TAZ extinction is shown under the histograms. **B)** Quantification of colonies in soft agar after 21 days. **C)** Caspase 3/7 activity. For all histograms, error bars indicate the SEM of at least three independent experiments. *P <0.05, **P <0.01, and ***P <0.001, using an ANOVA test followed by Dunnett’s test.
