# Supplementary figures and images for: MST1/Hippo promoter gene methylation predicts poor survival in patients with malignant pleural mesothelioma in the IFCT-GFPC-0701 MAPS Phase 3 trial
Source: Br J Cancer. 2019 Feb 11;120(4):387–97. doi: 10.1038/s41416-019-0379-8 (PMC6461894; doi:10.1038/s41416-019-0379-8)

## Slide 1
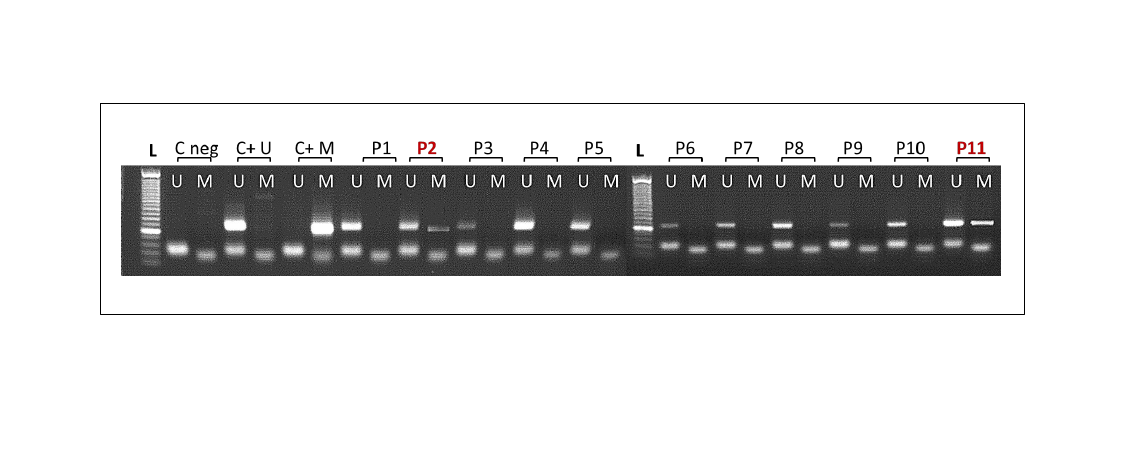

Supplement: Supplementary file 6 — FigureS1 [file 41416_2019_379_MOESM6_ESM.pptx]

## Slide 1
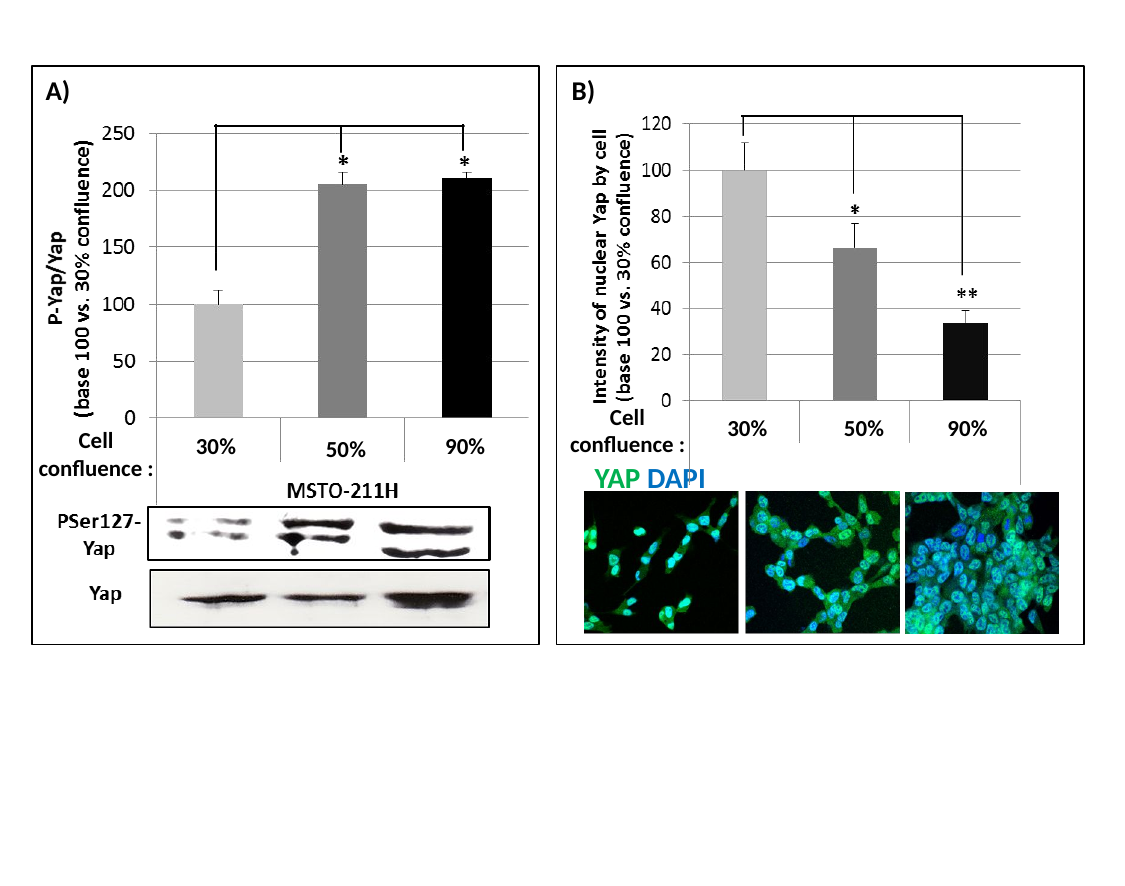

A)
B)
30%
50%
90%
YAP DAPI
30%
90%
50%
Cell confluence :
Cell confluence :

Supplement: Supplementary file 9 — FigureS4 [file 41416_2019_379_MOESM9_ESM.pptx]
