# Supplementary material for: MST1/Hippo promoter gene methylation predicts poor survival in patients with malignant pleural mesothelioma in the IFCT-GFPC-0701 MAPS Phase 3 trial
Source: Br J Cancer. 2019 Feb 11;120(4):387–97. doi: 10.1038/s41416-019-0379-8 (PMC6461894; doi:10.1038/s41416-019-0379-8)
Supplement: Supplementary file 7 — FigureS2 [file 41416_2019_379_MOESM7_ESM.pptx]

## Slide 1
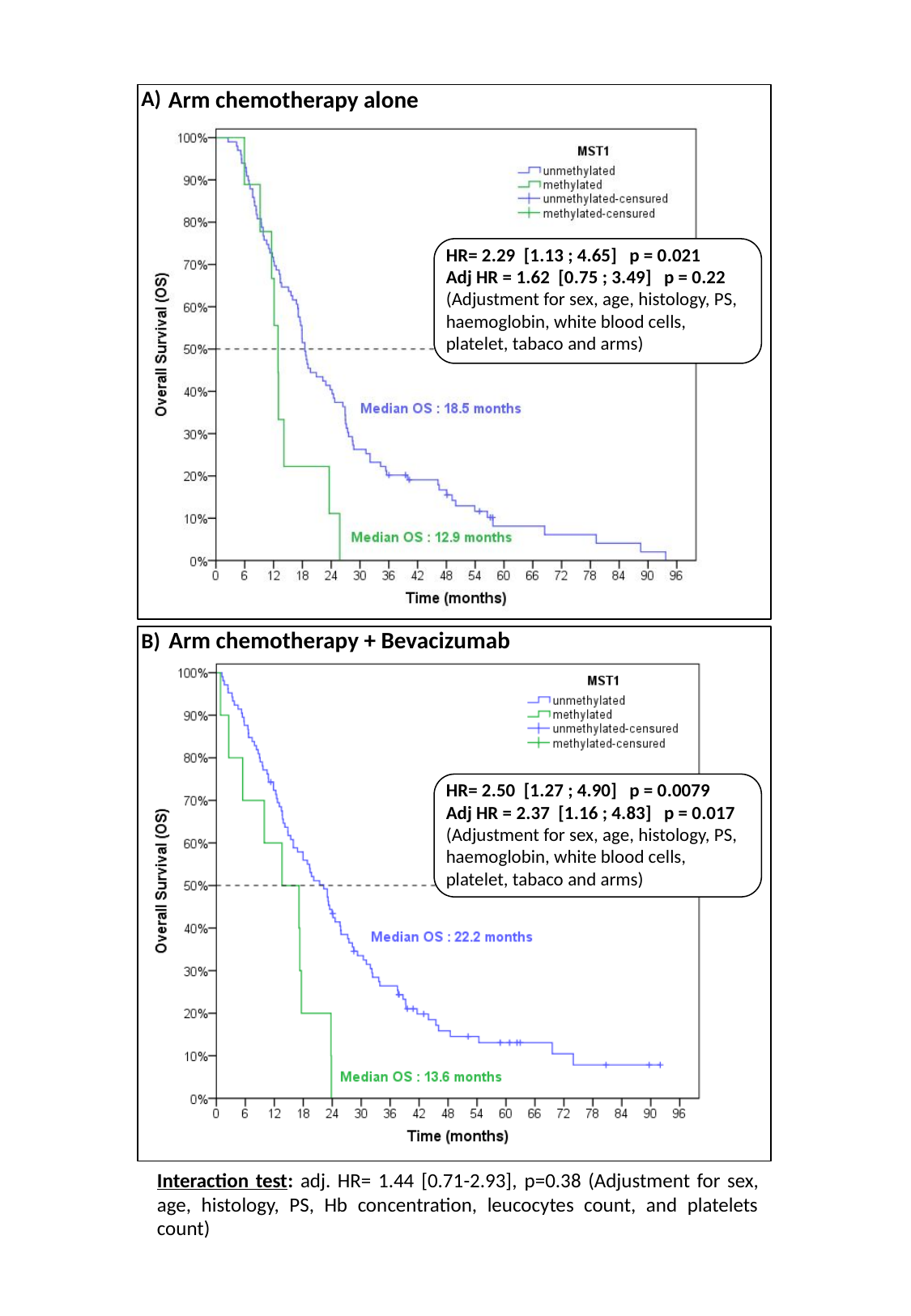

A)
Arm chemotherapy alone
HR= 2.29 [1.13 ; 4.65] p = 0.021
Adj HR = 1.62 [0.75 ; 3.49] p = 0.22
(Adjustment for sex, age, histology, PS, haemoglobin, white blood cells, platelet, tabaco and arms)
Arm chemotherapy + Bevacizumab
B)
HR= 2.50 [1.27 ; 4.90] p = 0.0079
Adj HR = 2.37 [1.16 ; 4.83] p = 0.017
(Adjustment for sex, age, histology, PS, haemoglobin, white blood cells, platelet, tabaco and arms)
Interaction test: adj. HR= 1.44 [0.71-2.93], p=0.38 (Adjustment for sex, age, histology, PS, Hb concentration, leucocytes count, and platelets count)
