# Supplementary material for: MST1/Hippo promoter gene methylation predicts poor survival in patients with malignant pleural mesothelioma in the IFCT-GFPC-0701 MAPS Phase 3 trial
Source: Br J Cancer. 2019 Feb 11;120(4):387–97. doi: 10.1038/s41416-019-0379-8 (PMC6461894; doi:10.1038/s41416-019-0379-8)
Supplement: Supplementary file 8 — FigureS3 [file 41416_2019_379_MOESM8_ESM.pptx]

## Slide 1
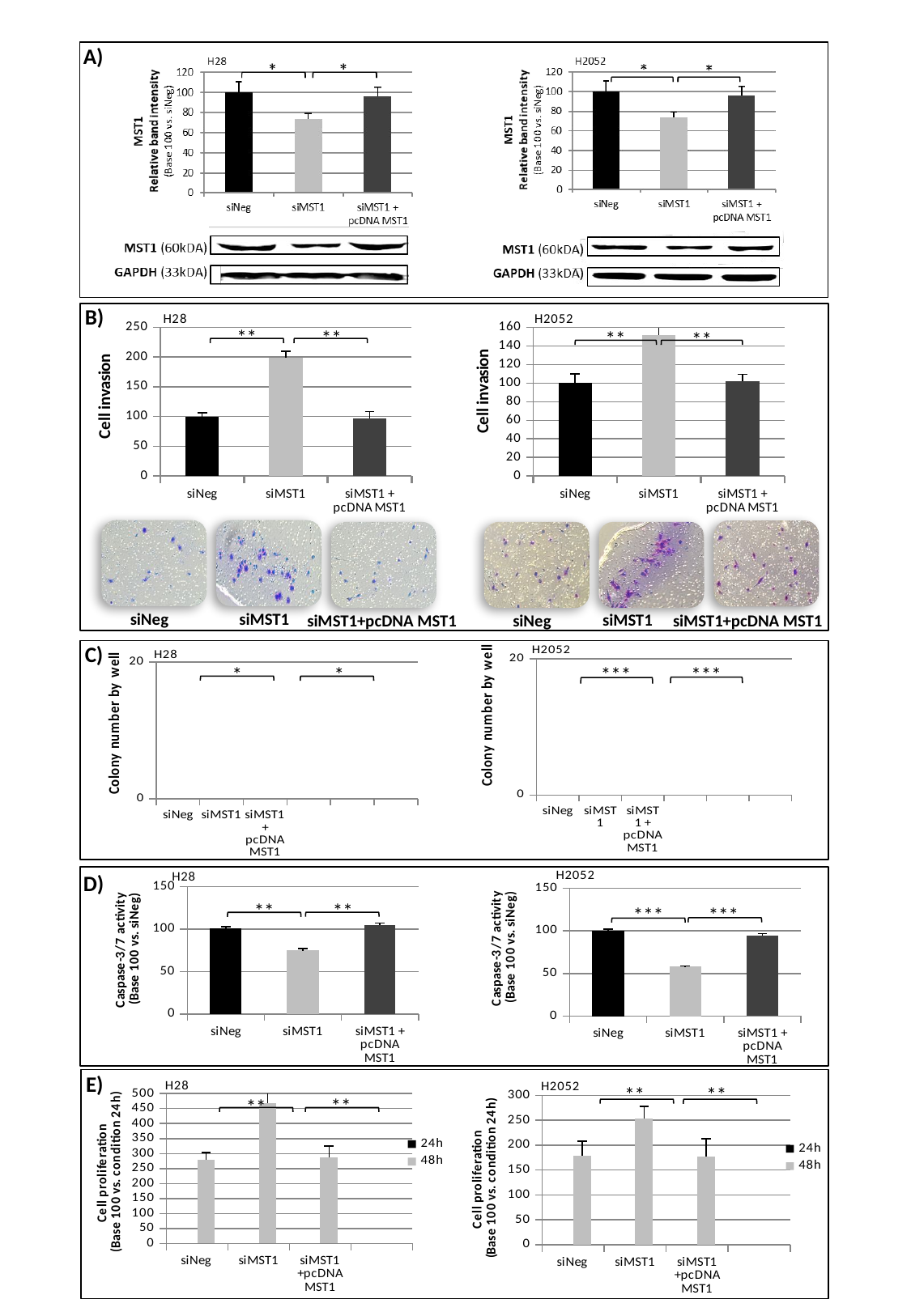

A)
B)
### Chart: H28
| Category | |
|---|---|
| siNeg | 100.0 |
| siMST1 | 199.10394265232978 |
| siMST1 + pcDNA MST1 | 97.34767025089604 |**
**
### Chart: H2052
| Category | |
|---|---|
| siNeg | 100.0 |
| siMST1 | 152.14670595105378 |
| siMST1 + pcDNA MST1 | 102.49155031763728 |**
**
siNeg
siMST1
siMST1
siMST1+pcDNA MST1
siNeg
siMST1+pcDNA MST1
C)
### Chart: H2052
| Category | |
|---|---|
| siNeg | 46.083333333333336 |
| siMST1 | 69.97222222222221 |
| siMST1 + pcDNA MST1 | 47.52777777777778 |***
***
### Chart: H28
| Category | |
|---|---|
| siNeg | 41.083333333333336 |
| siMST1 | 64.58333333333333 |
| siMST1 + pcDNA MST1 | 43.583333333333336 |*
*
### Chart: H2052
| Category | |
|---|---|
| siNeg | 100.0 |
| siMST1 | 57.73806605215186 |
| siMST1 + pcDNA MST1 | 94.88377902850978 |***
***
D)
### Chart: H28
| Category | |
|---|---|
| siNeg | 100.0 |
| siMST1 | 74.52262368617443 |
| siMST1 + pcDNA MST1 | 104.77227091612254 |**
**
E)
### Chart: H28
| Category | 24h | 48h |
|---|---|---|
| siNeg | 100.0 | 280.3326873385012 |
| siMST1 | 100.0 | 468.1922773837668 |
| siMST1 +pcDNA MST1 | 100.0 | 287.0078623349652 |**
**
**
### Chart: H2052
| Category | 24h | 48h |
|---|---|---|
| siNeg | 100.0 | 179.19020604882067 |
| siMST1 | 100.0 | 252.8332166199813 |
| siMST1 +pcDNA MST1 | 100.0 | 177.11742750502438 |**
