# Supplementary material for: MST1/Hippo promoter gene methylation predicts poor survival in patients with malignant pleural mesothelioma in the IFCT-GFPC-0701 MAPS Phase 3 trial
Source: Br J Cancer. 2019 Feb 11;120(4):387–97. doi: 10.1038/s41416-019-0379-8 (PMC6461894; doi:10.1038/s41416-019-0379-8)
Supplement: Supplementary file 10 — FigureS5 [file 41416_2019_379_MOESM10_ESM.pptx]

## Slide 1
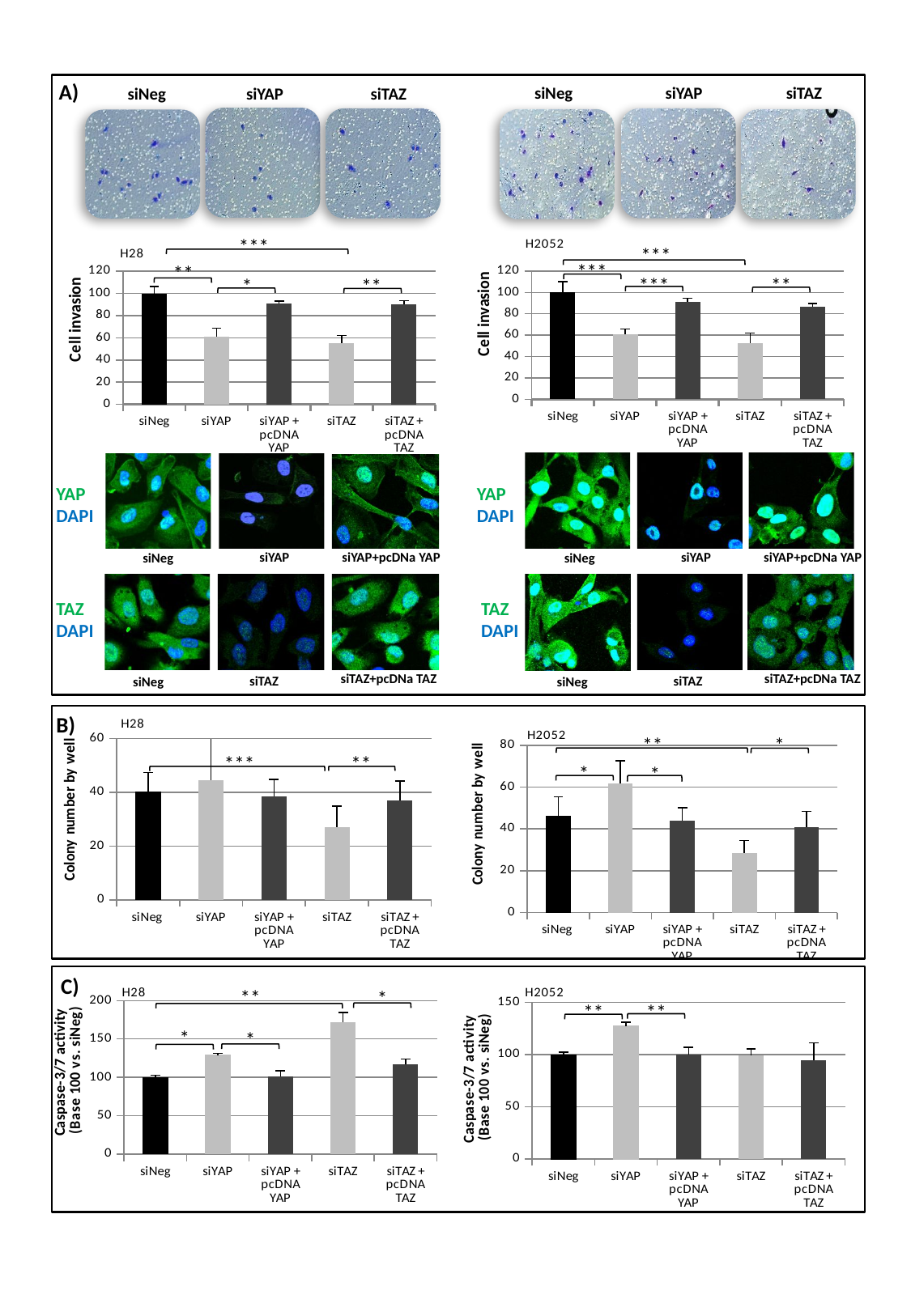

A)
siNeg
siYAP
siTAZ
siNeg
siTAZ
siYAP
### Chart: H28
| Category | |
|---|---|
| siNeg | 100.0 |
| siYAP | 61.0752688172043 |
| siYAP + pcDNA YAP | 90.67383512544802 |
| siTAZ | 55.11469534050179 |
| siTAZ + pcDNA TAZ | 90.00358422939068 |**
***
*
**
### Chart: H2052
| Category | |
|---|---|
| siNeg | 100.0 |
| siYAP | 60.76670913627435 |
| siYAP + pcDNA YAP | 91.42954469041426 |
| siTAZ | 52.497743802091634 |
| siTAZ + pcDNA TAZ | 86.7194440020527 |***
***
***
**
YAP
DAPI
YAP
DAPI
siNeg
siYAP
siYAP+pcDNa YAP
siNeg
siYAP
siYAP+pcDNa YAP
TAZ
DAPI
TAZ
DAPI
siTAZ+pcDNa TAZ
siTAZ+pcDNa TAZ
siTAZ
siTAZ
siNeg
siNeg
B)
### Chart: H28
| Category | |
|---|---|
| siNeg | 40.416666666666664 |
| siYAP | 44.61111111111111 |
| siYAP + pcDNA YAP | 38.583333333333336 |
| siTAZ | 27.166666666666668 |
| siTAZ + pcDNA TAZ | 37.083333333333336 |
***
**
### Chart: H2052
| Category | |
|---|---|
| siNeg | 46.083333333333336 |
| siYAP | 61.583333333333336 |
| siYAP + pcDNA YAP | 44.0 |
| siTAZ | 28.5 |
| siTAZ + pcDNA TAZ | 40.72222222222222 |*
*
**
*
C)
### Chart: H2052
| Category | |
|---|---|
| siNeg | 100.0 |
| siYAP | 127.43853407988712 |
| siYAP + pcDNA YAP | 100.38365787758725 |
| siTAZ | 98.8881448137045 |
| siTAZ + pcDNA TAZ | 94.23288554841372 |**
**
### Chart: H28
| Category | |
|---|---|
| siNeg | 100.0 |
| siYAP | 128.88347462711332 |
| siYAP + pcDNA YAP | 101.08797724118017 |
| siTAZ | 171.4559987299132 |
| siTAZ + pcDNA TAZ | 116.93883150920254 |**
*
*
*
